# Comparative Analysis of the Bibliographic Data Sources Dimensions and Scopus: An Approach at the Country and Institutional Levels

**DOI:** 10.3389/frma.2020.593494

**Published:** 2021-01-22

**Authors:** Vicente P. Guerrero-Bote, Zaida Chinchilla-Rodríguez, Abraham Mendoza, Félix de Moya-Anegón

**Affiliations:** ^1^ Departamento de Información y Comunicación, Universidad de Extremadura, Badajoz, Spain; ^2^ Instituto de Políticas y Bienes Públicos (IPP), Consejo Superior de Investigaciones Científicas (CSIC), Madrid, Spain; ^3^ Facultad de Ingeniería, Universidad Panamericana, Zapopan, Mexico; ^4^ SCImago Research Group, Granada, Spain

**Keywords:** Dimensions, Scopus, bibliographic data sources, database coverage, research evaluation, scientometrics, bibliometrics

## Abstract

This paper presents a large-scale document-level comparison of two major bibliographic data sources: Scopus and Dimensions. The focus is on the differences in their coverage of documents at two levels of aggregation: by country and by institution. The main goal is to analyze whether Dimensions offers as good new opportunities for bibliometric analysis at the country and institutional levels as it does at the global level. Differences in the completeness and accuracy of citation links are also studied. The results allow a profile of Dimensions to be drawn in terms of its coverage by country and institution. Dimensions’ coverage is more than 25% greater than Scopus which is consistent with previous studies. However, the main finding of this study is the lack of affiliation data in a large fraction of Dimensions documents. We found that close to half of all documents in Dimensions are not associated with any country of affiliation while the proportion of documents without this data in Scopus is much lower. This situation mainly affects the possibilities that Dimensions can offer as instruments for carrying out bibliometric analyses at the country and institutional level. Both of these aspects are highly pragmatic considerations for information retrieval and the design of policies for the use of scientific databases in research evaluation.

## Introduction

As new multidisciplinary scientific bibliographic data sources are coming onto the market, there is growing interest in comparative studies looking at aspects of the coverage they offer. Scholarly databases have begun to play an increasingly important role in the academic ecosystem. There are several reasons for this, including burgeoning competitiveness in research, greater availability of data, and the need to justify the use of public funds. This context has driven the diversification of evaluations of publication and citation data use cases as well as of research use cases that have not been met by existing scholarly databases (Hook et al., 2018). Since bibliometric methods are used in multiple areas for a variety of purposes, especially research evaluation, the results they provide may vary depending on the representativeness of the database used (Mongeon and Paul-Haus, 2016; Huang et al., 2020). The new data sources can offer several benefits for research evaluators because they may have better coverage or have capabilities that make them a better fit for a given impact evaluation task, and they can reduce the cost of evaluations and make informal self-evaluations of impact possible for researchers who would not pay to access that kind of data (Thelwall, 2018). Given the potential value of these data sources for research evaluation, it is important to assess their key properties to better understand their strengths and weaknesses, in particular, to decide whether their data is sufficient in volume, completeness, and accuracy to be useful for scientists, policymakers, and other stakeholders.

Traditionally, the only homogeneous record of published research available when funders and governments sought additional information to help them make evidence-driven decisions was the Web of Science (WoS). The appearance of the Scopus database (Baas et al., 2020) and Google Scholar in 2004 as “competitors” to WoS, providing metadata on scientific documents and on citation links between these documents, led to an immense quantity of studies focused on comparative analyses of these other new bibliographic sources, the basic intention being to look for novel bibliometric opportunities that these tools might bring to the academic community and policymakers.

At that time, it appeared that Scopus and WoS had entered into head-on competition (Pickering, 2004), and any comparison of them called for the utmost care and methodological consistency. One large-scale comparison at the journal level was done using Ulrich’s directory as the gold standard by Moya-Anegón et al. (2007). The results outlined a profile of Scopus in terms of its coverage by areas—geographic and thematic—and the significance of peer-review in the publications. Both of these aspects are of highly pragmatic significance for policymakers and the users of scientific databases. Years later, Mongeon and Paul-Haus (2016) revisited the issue and compared the coverage of WoS and Scopus to examine whether preexisting biases (such as language, geography, and theme) were still to be found in Scopus. They concluded that some biases still remained in both databases and stated that this should be taken into account in assessing scientific activities. For example, most languages and countries are underrepresented, which contributes to the known lack of visibility of research done in some countries. Hence, when using bibliometric methods for research evaluation, it is important to understand what each tool has to offer and what its limitations are and to choose the right tool for the task at hand before drawing conclusions for research evaluation purposes (Mongeon and Paul-Haus, 2016).

Google Scholar appeared to be an alternative to WoS and Scopus, but its suitability for research evaluation and other bibliometric analyses was called strongly into question. For a comprehensive review of this data source in research evaluation, we would refer to Martín-Martín et al. (2018a) and Martín-Martín et al. (2020).

At the beginning of 2018, Digital Science launched Dimensions, a new integrated database covering the entire research process from funding to research, from publishing results through attention, both scholarly and beyond, to commercial applications and policymaking, consistently matched in multiple dimensions (Adams et al., 2018). This new scholarly data source was created to overcome significant constraints of the existing databases. It sought to understand the research landscape through the lens of publication and citation data and help the academic community to formulate and develop its own metrics that can tell the best stories and give the best context to a line of research (Bode et al., 2019).

Previous studies have compared data quality between Dimensions and other data sources in order to evaluate its reliability and validity (Bornmann, 2018; Martín-Martín et al., 2018; Thelwall, 2018; Visser et al., 2020). Most of them have focused on publication and citation in specific thematic fields, but few of them have taken a global perspective. The findings of these studies in the field of Food Science show Dimensions to be a competitor to WoS and Scopus in making nonevaluative citation analyses and in supporting some types of formal research evaluations (Thelwall, 2018). Similarly, Martín-Martín et al. (2018b) conclude that Dimensions is a clear alternative for carrying out citation studies, being capable of rivalling Scopus. But the reliability and validity of its field classification scheme were questioned. This scheme is not based on journal classification systems as it is in WoS or Scopus, but on machine learning. This feature makes it desirable to undertake large-scale investigations in future studies to ensure that metrics such as the field-normalized citation scores presented in Dimensions and calculated based on its field classification scheme are indeed reliable (Bornmann, 2018).

A large-scale comparison of five multidisciplinary bibliographic data sources, including Dimensions and Scopus, was carried out recently by Visser et al. (2020). They used Scopus as the baseline for comparing and analyzing not just the different coverage of documents over time by document type and discipline but also the completeness and accuracy of the citation links. The results of this comparison shed light on the different types of documents covered by Dimensions but not by Scopus. These are basically meeting abstracts and other short items that do not seem to make a very substantial contribution to science. The authors concluded that differences between data sources should be assessed in accordance with the purpose for which the data sources are used. For example, it may be desirable to work within a more restricted universe of documents, such as a specific thematic field or a specific level of aggregation. This is the case with the study of Huang et al. (2020) which compared WoS, Scopus, and Microsoft Academic and their implications for the robustness of university rankings.

The present communication extends previous comparisons of Scopus by expanding the study set to include distinct levels of aggregation (by country and by institution) across a larger selection of characteristics and measures. A particular aim is to inquire closely into just how balanced Dimensions’ coverage is compared with that of the Scopus database.

### Objectives/Research Questions

The goal of this study was to compare Dimensions’ coverage with that of Scopus at the geographic and institutional levels. The following research questions were posed:

(1) How comprehensive is Dimensions’ coverage compared with that of Scopus in terms of documents?
(2) Are the distributions of publications by country and by institution in Dimensions comparable with those in Scopus?
(3) Are Dimensions’ citation counts by country and by institution interchangeable with those of Scopus in the sense of their being strongly correlated?
(4) Is Dimensions a reliable new bibliometric data source at the country and institutional levels?

## Material and Methods

Scopus is a scientific bibliography database created by Elsevier in 2004 (Hane, 2004; Pickering, 2004) which has been extensively characterized (Moya-Anegón et al., 2007; Archambault et al., 2009; Leydesdorff et al., 2010) and used in scientometric studies (Gorraiz et al., 2011; Jacso, 2011; Guerrero-Bote and Moya-Anegón, 2015; Moya-Anegón et al., 2018). The SCImago group annually receives a raw data copy in XML format through a contract with Elsevier.

In 2018, Digital Science published the Dimensions database with scientific publications and citations, grants, patents, and clinical trials (Hook et al., 2018; Herzog et al., 2020). Since then, there has been characterization published of it (Bornmann, 2018; Harzing, 2019; Visser et al., 2020). In the present study, we shall only consider the scientific publications.

Bibliographic databases often give bibliometric studies problems with author affiliations which usually do not include standardized names of institutions. One of the improvements that Dimensions incorporates is the mapping of author affiliations in documents to an entity list for organizations involved in research. This is the GRID (Global Research Identifier Database) system (Hook et al., 2018). This mapping is not an addition to but a replacement for author affiliations. If this mapping is rigorous and complete, it is an important improvement. But if the list of organizations or the mapping is incomplete, this could be a major problem because there would be loose documents without any possibility of associating them with institutions or countries, thus leaving the output of the institutions and countries affected incomplete.

The SCImago group has had the possibility of downloading a copy of Dimensions in Json format through an agreement with Dimensions Science.

From the Scopus and Dimensions data of April 2020, the SCImago group created a relational database for internal use that allows for massive computation operations that would otherwise be unfeasible.

### Matching

For the analysis that was an objective of this study, it was necessary to implement a matching procedure between the Dimensions and Scopus databases. To this end, we applied the method developed in the SCImago group to match PATSTAT NPL references with Scopus documents (Guerrero-Bote et al., 2019). This method has two phases: a broad generation of candidate pairs, followed by a second phase of pair validation.

In this case, a modification was made, similar to that in Visser et al. (2020), in which not all the candidate pairs were generated at the same time. Instead, once there was a set of candidate pairs, a validation procedure was applied, accepting as valid the matches that exceeded a certain threshold. This reduced the combinatorial variability of the following generations of candidates. The pairs that did not exceed the threshold were not discarded but were saved in case at the end they were unpaired and were those with the greatest similarity.

In more detail, our procedure began with the normalization of the fields to facilitate pairing, although, unlike Visser et al. (2020), we did not stay exclusively with the numerical values of the volume, issue, or pages because at times those fields do not contain numerical values. This is the case with journals such as PLOS One or Frontiers, for instance.

Then we started to generate candidate pairs in phases. The phases were centered on the following conditions:

(1) One of these conditions:
  (1) Same year of publication, title with a high degree of similarity, and the same DOI.
  (2) Same year of publication, title with a high degree of similarity, and the same authors.
  (3) Same year of publication, title, and first author.
(2) One of these conditions:
  (1) Same year of publication and DOI.
  (2) Same year of publication, source (journal, proceeding, etc.), volume, and pages.
  (3) Same year of publication and coincidence in the first or last 20 characters of the title.
  (4) Same year of publication and authors.
  (5) Same year of publication and source.

As can be seen, there are conditions that include some previous phases. However, it should be borne in mind that each candidate pair generation phase is followed by a validation phase. So the first phases are quite specific; they generate a relatively small number of candidate pairs, most of which are accepted and come to constitute the majority of the definitively matched pairs. In this way, the lists of documents waiting to be matched are reduced, allowing for broader searches in the following phases without greatly increasing the computational cost. Logically, the percentage of success in the candidate pairs decreases from phase to phase.

For validation, all the reference's data were compared: DOI, year of publication, authors, title, publication, volume, issue, and pages. The last three were compared both numerically and alpha-numerically. The comparison of each field generated a numerical score corresponding to the number of matching characters with some adjustments, for which the Levenshtein^1^ distance was used as in Guerrero-Bote et al. (2019) and Visser et al. (2020). Once the coincidence score had been calculated in each field, we took the product to get the total score. The individual scores by field never have a zero value because that would mean the total score would be zero. In case of noncoincidence, the field score may be unity if the field is considered to be nonessential, 0.75 if it is considered to be important, etc. In either of the databases, the fields of some records may be empty. With this process, coincidence in several fields increases the total score geometrically rather than arithmetically.

Once the candidate pairs of a phase have been validated, we take as matched the pairs that obtain a total score greater than 1,000, and in which neither the Scopus nor the Dimensions record scores higher with any other pair. The total score threshold of 1,000 was set after sampling and verifying that under these conditions no mismatched pair was found.

Once the 5 phases had been carried out, a repechage operation was initiated for the rejected candidate pairs. This accepted pairs in which both components obtained a lower score in the rest of the pairs, down to a total score of 50. Also accepted were those in which the score was greater than 300, but one of the components had another pair with exactly the same score. This latter was done because both databases contain some duplicated records.

## Results

### The Results of Matching

The general results are given in Table 1. It is true that, even though our study includes more years than that of Visser et al. (2020), it gives fewer matched documents for the period 2008–2017.

**TABLE 1:** Overall results of the linking procedure.

Year	Total matches	% change	% matches Scopus	% matches Dimensions	Total Scopus	% change	Total Dimensions	% change
2003	1,102,377	—	70.01	56.05	1,571,723	—	1,966,869	—
2004	1,175,774	6.66	69.61	54.49	1,686,413	7.30	2,157,735	9.70
2005	1,295,013	10.14	67.34	57.17	1,920,131	13.86	2,265,278	4.98
2006	1,406,239	8.59	69.59	56.87	2,019,216	5.16	2,472,883	9.16
2007	1,485,168	5.61	69.95	53.45	2,124,118	5.20	2,778,498	12.36
2008	1,566,745	5.49	70.37	56.74	2,227,050	4.85	2,761,246	−0.62
2009	1,665,294	6.29	71.17	56.73	2,342,897	5.20	2,935,302	6.30
2010	1,768,496	6.20	71.78	57.65	2,465,117	5.22	3,067,425	4.50
2011	1,902,640	7.59	72.52	54.52	2,625,462	6.50	3,489,937	13.77
2012	1,986,358	4.40	72.13	55.19	2,755,115	4.94	3,599,181	3.13
2013	2,085,792	5.01	72.62	54.05	2,874,153	4.32	3,859,025	7.22
2014	2,147,442	2.96	73.6	52.77	2,922,477	1.68	4,069,795	5.46
2015	2,182,437	1.63	75.52	52.04	2,891,116	−1.07	4,193,437	3.04
2016	2,259,015	3.51	75.54	51.62	2,990,795	3.45	4,376,598	4.37
2017	2,357,244	4.35	75.22	49.94	3,133,127	4.76	4,720,253	7.85
2018	2,533,236	7.47	79.15	50.33	3,190,038	1.82	5,033,439	6.63
2019	2,659,664	4.99	81.03	51.60	3,270,544	2.52	5,154,828	2.41
Total	31,578,934	—	73.39	53.61	43,009,492	—	5,8,901,729	—

The number of matched pairs grows from year to year, and in Scopus, the percentage of matches also grows. This is not the case for Dimensions, however, due to the great growth this database experienced from year to year.

In summary, Dimensions’ coverage is more than 25% greater than Scopus’s, although there is a significant overlap in coverage between the two data sources. Almost three-quarters of the Scopus documents and more than half of the Dimensions documents match. The question now is to see if these percentage differences are maintained at levels of grouping of lower rank (countries and institutions).

The percentage of matching in Scopus by document type is presented in Table 2. The greatest percentages are in articles, reviews, letters, conference proceedings, errata, editorials, book chapters, short surveys, etc. (We have not listed some document types due to their low output.) For the primary output (articles, reviews, conference proceedings, and short surveys), the matching is over 75%.

**TABLE 2:** Scopus matching percentages by most frequent document type.

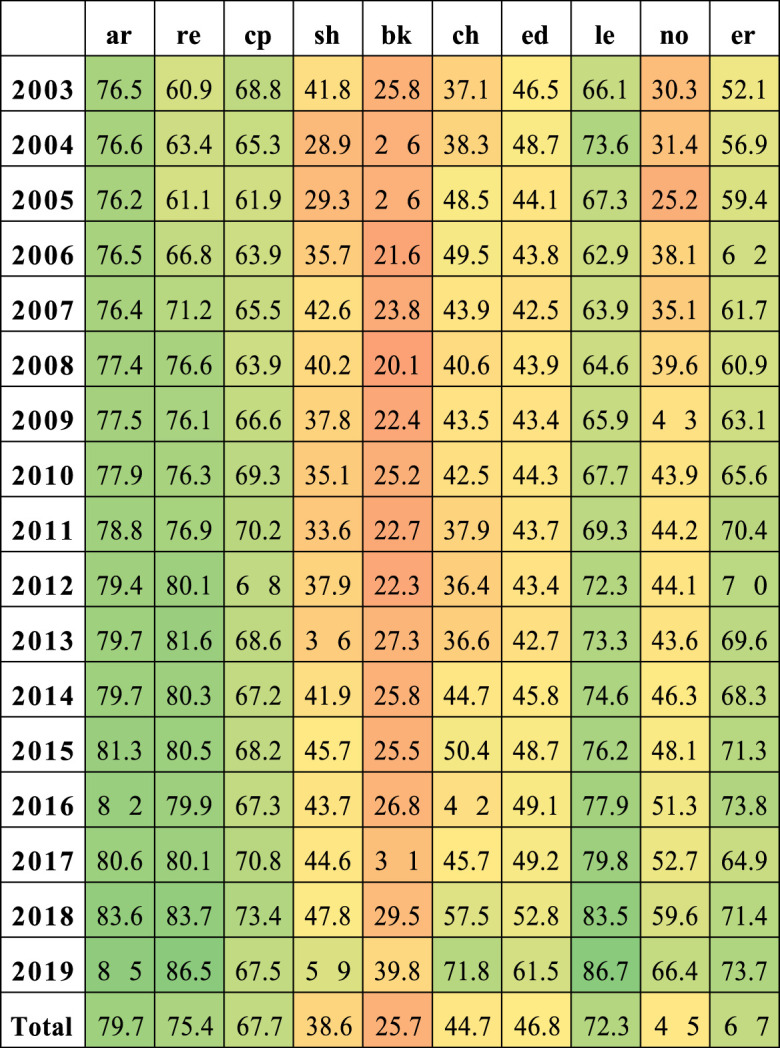

AR, articles; RE, reviews; CP, conference proceedings; SH, short survey; BK, book chapter; ED, editorial; LE, letters; NO, note; ER, erratum.

Table 3 presents the same information, but for Dimensions. Articles and conference proceedings are the most matched types.

**TABLE 3:** Dimensions matching percentages by document type.

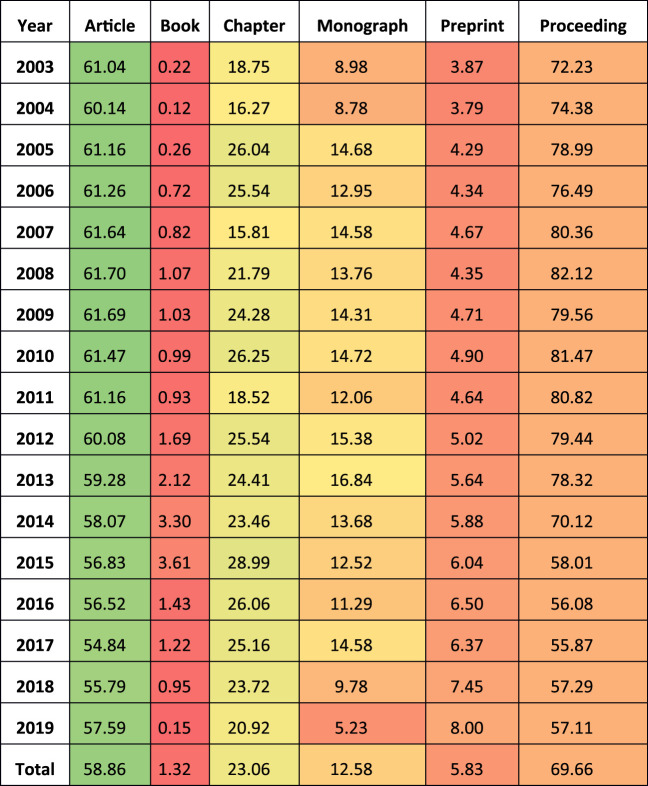

**FIGURE 1:**
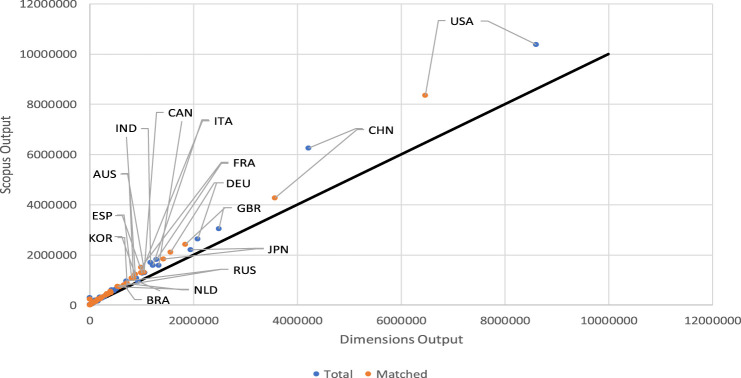
Scatter plot of the total and matched Dimensions/Scopus output by country.

Figure 1 shows that the total and matched output distributed by country is systematically greater in Scopus than in Dimensions. The solid line represents the ideal positions of the countries if they had the same output in Scopus and Dimensions. It is noticeable at a glance that most countries appear above the solid line in the graph, indicating that the Scopus output by country tends to be greater than the Dimensions output.

**FIGURE 2:**
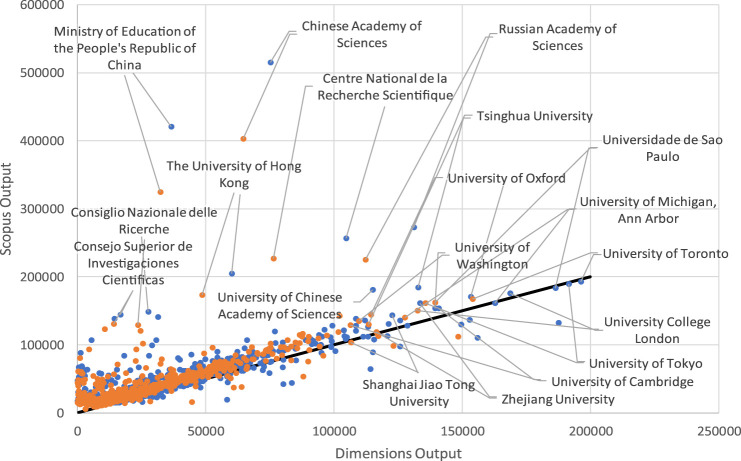
Scatter plot of the total and matched Dimensions/Scopus output by institution.

Figure 2 shows the relationship of the output by institution between Dimensions and Scopus. The solid line represents the positions of the institutions if they had the same output in both databases. It is again noticeable at a glance that most institutions are above the solid line, indicating that there are more institutions with more output in Scopus than in Dimensions.

**FIGURE 3:**
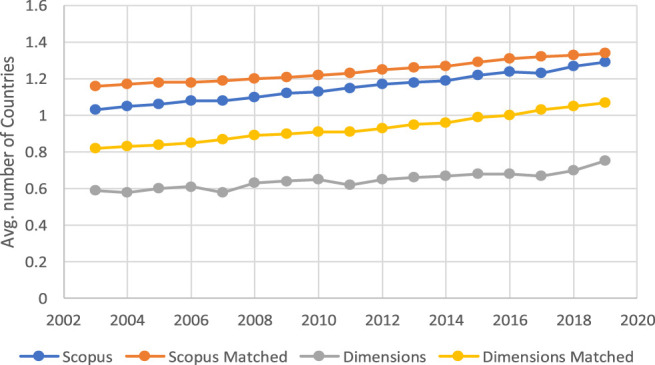
Evolution of the average number of countries per document in Scopus and Dimensions in total and in the matched subsets.

Figure 3 allows one to analyze the evolution of the average number of countries whose institutions correspond to the author's affiliations in the documents present in one or the other database. What most stands out in this graph is the difference between the two databases. The two sets of evolution should be very similar, and yet they are not. These differences remain stable over time and need to be confirmed with the data representing the evolution of the number of institutions that appear in the author's affiliations.

**FIGURE 4:**
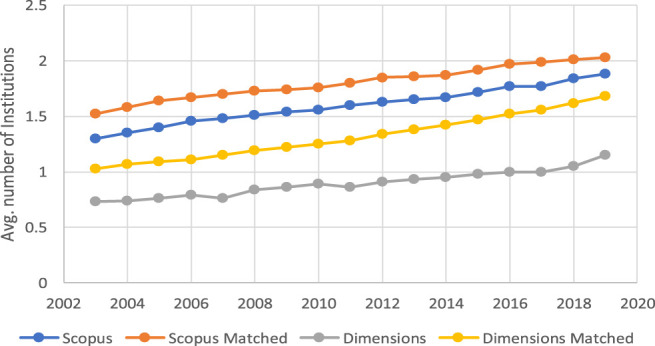
Evolution of the average number of institutions per document in Scopus and Dimensions in total and in the matched subsets.

Figure 4 confirms, from the institutional perspective, the evolution of the average of institutions per document in the two databases and in the matched documents. The two sets of evolution reveal the average of institutional affiliations associated with the items in the four subsets of the two data sources. As can be seen, the comparison between the two graphical representations is consistent.

**FIGURE 5:**
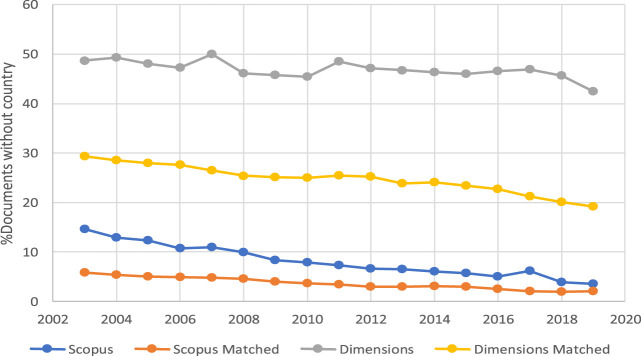
Evolution of the annual percentage of items without country in the four subsets of documents belonging to Dimensions and Scopus.

In order to check the influence of documents without a country on the averages presented in Figures 3, 4, Figure 5 shows the evolution of the percentage of items in the four subsets of documents that do not record any country for some reason. As can be seen in the figure, these percentages have a downwards trend over the years in the different subsets of documents, and the order of the curves is contrary to that in Figures 3, 4, which is consistent from the perspective of data interpretation.

In general terms, one can say that the information about institutional affiliations that allows documents to be discriminated by country and institution has greater completeness in Scopus than in Dimensions. The case is similar when analyzing this same situation from the perspective of the matched documents. In terms of temporal evolution, despite the positive trend in the number of countries and institutions associated with the items in both databases, the difference between the two sources in this regard tends to be maintained over time.

A more detailed characterization of the Dimensions documents where no country affiliation data is available is provided in Table 4. The distribution of document types shows that there are distinct document types affected by this situation.

**TABLE 4:** Distribution of document types where no country affiliation data is available.

	Article				Book				Chapter			
	Total	Yes	No	%	Total	Yes	No	%	Total	Yes	No	%
2003	1,594,777	847,696	747,081	46.85	5,043	0	5,043	100.00	178,482	60,138	118,344	66.31
2004	1,710,220	921,384	788,836	46.12	5,151	0	5,151	100.00	236,614	65,718	170,896	72.23
2005	1,790,924	969,678	821,246	45.86	6,133	0	6,133	100.00	227,321	75,457	151,864	66.81
2006	1,929,725	1,072,799	856,926	44.41	6,359	0	6,359	100.00	264,507	89,235	175,272	66.26
2007	2,008,313	1,132,294	876,019	43.62	7,561	0	7,561	100.00	471,557	99,410	372,147	78.92
2008	2,108,438	1,193,113	915,325	43.41	7,471	0	7,471	100.00	324,582	112,560	212,022	65.32
2009	2,210,781	1,272,372	938,409	42.45	8,172	0	8,172	100.00	350,024	125,995	224,029	64.00
2010	2,323,835	1,339,765	984,070	42.35	9,405	0	9,405	100.00	312,837	115,127	197,710	63.20
2011	2,555,664	1,483,092	1,072,572	41.97	10,373	0	10,373	100.00	503,972	123,081	380,891	75.58
2012	2,742,694	1,607,802	1,134,892	41.38	12,258	0	12,258	100.00	425,005	127,446	297,559	70.01
2013	2,938,822	1,714,338	1,224,484	41.67	12,181	0	12,181	100.00	474,432	142,900	331,532	69.88
2014	3,122,791	1,811,016	1,311,775	42.01	12,146	0	12,146	100.00	477,231	154,709	322,522	67.58
2015	3,266,544	1,884,432	1,382,112	42.31	13,043	0	13,043	100.00	414,925	154,310	260,615	62.81
2016	3,430,797	1,944,920	1,485,877	43.31	14,272	0	14,272	100.00	377,731	155,480	222,251	58.84
2017	3,652,464	2,076,024	1,576,440	43.16	15,196	0	15,196	100.00	440,965	167,278	273,687	62.07
2018	3,863,842	2,276,994	1,586,848	41.07	17,308	0	17,308	100.00	502,279	182,953	319,326	63.58
Growth rate	142.28	168.61	112.41	−12.33	243.21	0.00	243.21	0.00	181.42	204.22	169.83	−4.12

	Monograph				Preprint				Proceeding			
	Total	Yes	No	%	Total	Yes	No	%	Total	Yes	No	%
2003	12,579	1,146	11,433	90.89	48,039	9,309	38,730	80.62	127,949	91,072	36,877	28.82
2004	12,827	1,083	11,744	91.56	50,798	8,041	42,757	84.17	142,125	97,487	44,638	31.41
2005	12,593	506	12,087	95.98	55,872	8,847	47,025	84.17	172,435	121,144	51,291	29.75
2006	12,339	537	11,802	95.65	61,009	9,552	51,457	84.34	198,944	131,960	66,984	33.67
2007	14,005	736	13,269	94.74	68,801	10,669	58,132	84.49	208,261	144,690	63,571	30.52
2008	14,403	875	13,528	93.92	75,226	11,916	63,310	84.16	231,126	168,755	62,371	26.99
2009	18,709	1,066	17,643	94.30	84,053	13,431	70,622	84.02	263,563	177,408	86,155	32.69
2010	20,997	1,429	19,568	93.19	93,239	14,628	78,611	84.31	307,112	204,415	102,697	33.44
2011	21,356	1,719	19,637	91.95	103,214	16,121	87,093	84.38	295,358	172,933	122,425	41.45
2012	28,405	1,777	26,628	93.74	114,152	18,305	95,847	83.96	276,667	147,489	129,178	46.69
2013	39,622	2,484	37,138	93.73	120,577	17,205	103,372	85.73	273,391	178,389	95,002	34.75
2014	33,389	3,073	30,316	90.80	125,340	18,374	106,966	85.34	298,898	196,218	102,680	34.35
2015	31,467	3,346	28,121	89.37	134,199	19,462	114,737	85.50	333,259	201,161	132,098	39.64
2016	38,941	4,478	34,463	88.50	144,704	21,845	122,859	84.90	370,153	211,794	158,359	42.78
2017	41,935	4,854	37,081	88.42	164,527	28,763	135,764	82.52	405,166	227,657	177,509	43.81
2018	37,792	5,292	32,500	86.00	193,204	37,024	156,180	80.84	419,014	232,398	186,616	44.54
Growth rate	200.44	361.78	184.26	−5.38	302.18	297.72	303.25	0.27	227.49	155.18	406.05	54.53

**FIGURE 6:**
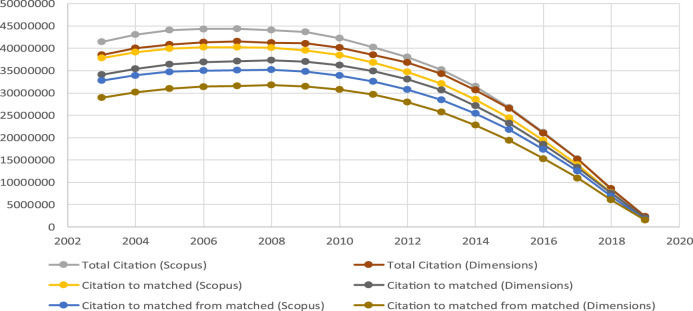
Citations by cited year.

**FIGURE 7:**
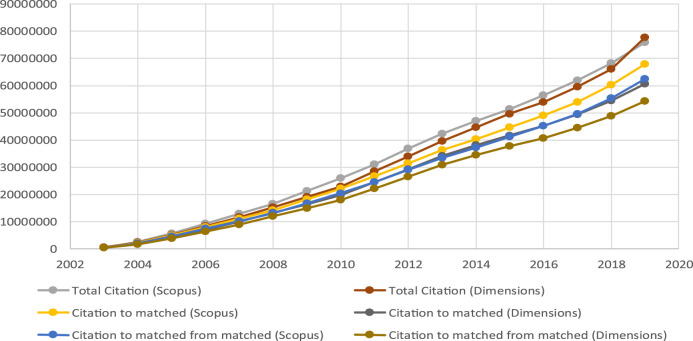
Citations by citing year.

Using as a basis the citation data (Figure 6), it is easy to see that, both for total documents and for matched documents, the volume of citations in Scopus is in all cases greater than that of Dimensions, as noted previously by Visser et al. (2020). The case is similar when the problem is analyzed from the point of view of the citing date (Figure 7).

**FIGURE 8:**
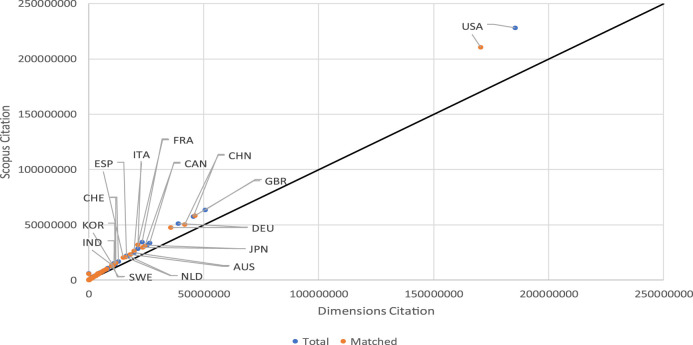
Relationship between total citations and matched documents by country.

**FIGURE 9:**
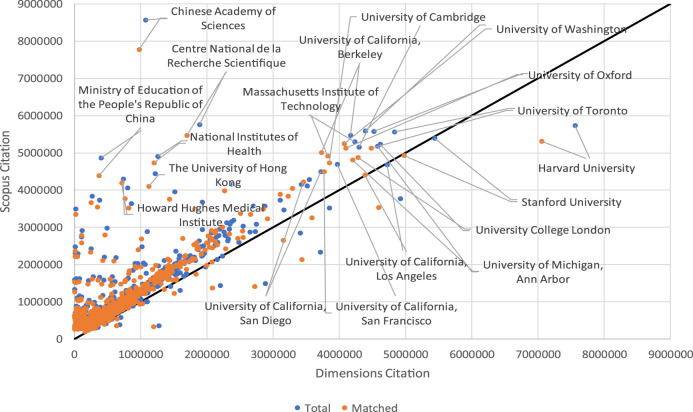
Relationship between total citations and matched documents by institution.

When the citations of the documents in the two databases are distributed by country, one observes that all of them, regardless of the size of their output, accumulate more citations in the Scopus database than in the Dimensions one. Figure 8 shows that both total citations and those of matched documents are consistently greater in Scopus than in Dimensions for all countries. The case is similar when the distribution of citations is by institution in the period of observation. The distribution of citations by institution is also greater in Scopus than in Dimensions in more than 97% of the cases. Figure 9 shows very clearly how just a small group of institutions lies below the straight line, and these conform to the 2.5% of cases that have more citations in Dimensions than in Scopus.

## Discussion

Our starting hypothesis was that the difference in overall coverage between the two databases should be similar in general terms when the total set of documents was fragmented into smaller levels of aggregation. From our perspective, it is important that overall coverage levels be maintained on average when the source is split into smaller groupings (countries or institutions, for example) in order to guarantee the bibliometric relevance of the source. For this reason, we continued along the path begun by other workers trying to deepen the comparative analysis of the coverage of the two sources.

Our first conclusion is that, for reasons that have to do with the data structures themselves, the two sources have notable differences in coverage at the level of countries and institutions, with a tendency for there to be greater coverage at those levels in Scopus than in Dimensions. This is even though what was to be expected would have been the opposite, given the overall differences in coverage between the two sources.

Second, despite the fact that Dimensions has a larger raw coverage of documents than Scopus, close to half of the documents in Dimensions lack country or institutional affiliation information, which means that when documents are aggregated by country or institutional affiliation, Scopus systematically provides more documents/citations than Dimensions. In 2014, Dimensions started working on the problem of creating an entity list for organizations to provide a consistent view of an organization within one content source, but also across the various different types of content. This was the GRID (Global Research Identifier Database) system. At that time, a set of policies about how to handle the definition of a research entity was developed.^2^ At the time of writing, GRID contains 98,332 unique organizations, for which the data has been curated and each institution assigned a persistent identifier. This set of institutions represents an international coverage of the world’s leading research organizations, indexing 92% of funding allocated globally. It is clear, however, that the repeated differences between Scopus and Dimensions in output and citation are related to the fact that Dimensions’ method of linking institutional affiliations to GRID, while a promising idea, is still a work in progress. In overall terms, currently, it limits linkages of item with countries and institutions. This situation mainly affects the possibilities that the two sources can offer as instruments for carrying out bibliometric analyses.

As Bode et al. (2019) point out in Dimensions’ Guide v.6 (p. 3), “Linked and integrated data from multiple sources are core to Dimensions. These matchings are data driven, then, the content and enrichment pipeline is as automated as possible. However, while an automated approach allows us to offer a more open, free approach it also results in some data issues, which we will continue to have to work on and improve.” This is advisable for both the publications and citation links because, as Visser et al. (2020) noted, “Dimensions incorrectly has not identified citation links. Hence, this data source fails to identify a substantial number of citation links” (p. 20). Dimensions also has the limitation that it does not provide data for references that have not been matched with a cited document (p. 23).

The results described should help fill the gap in exploring differences between Scopus and Dimensions at the country and institutional levels. Figure 5 appears to be the main cause that explains most of the other results. Most of the other results in this manuscript are an effect or consequence of this. This should allow a profile of Dimensions to be outlined in terms of its coverage by different levels of aggregation of its publications in comparison with Scopus. Both of these aspects are highly pragmatic considerations for bibliometric researchers and practitioners, in particular for policymakers who rely on such databases as a principal criterion for research assessment (hiring, promotion, and funding).

At the country level, this study has shown that not all articles had complete address data. Even though there was a decreasing trend over time in the number of documents with no country information in the address data, in 2018 still more than 40% of documents in Dimensions remained without a country. Given the size of the data source and its goal in the scientific market, missing information of the country in the affiliation data has important implications at all levels of aggregation and analysis. Thus, Dimensions does not currently appear to be a reliable data source with which to define and evaluate the set of output at the country level.

At the institutional level, according to Huang et al. (2020), “Universities are increasingly evaluated on the basis of their outputs which are often converted to rankings with substantial implications for recruitment, income, and perceived prestige.” The present study has shown that Dimensions does not record all institutional affiliation of the authors, which has implications for metrics and rankings at the institutional scale. In this case, it seems advisable to integrate diverse data sources into any institutional evaluation framework (Huang et al., 2020).

We have not been comparing document types but presenting results derived from the matching procedure. As in Visser et al. (2020), we found that there were many articles in Dimensions for which there was no matching document in our matching procedure. This is because it seems that any document published in a journal is classified as an article in Dimensions.

Finally, as in previous studies examining data sources’ coverage (Moya-Anegón et al., 2007), to very briefly conclude and with possible future bibliometric studies in mind, the above considerations conform to an important part of the context of scientific output and evaluation and should be taken into account so as to avoid bias in the comparison of research results in diverse domains or at different aggregation levels. All data sources suffer from problems of incompleteness and inaccuracy of citation links (Visser et al., 2020, p. 23), and GRID is not yet perfect and never will be (Bode et al., 2019, p. 6). But we are confident that studies like the present will help to improve this tool and the data in the near future.

## Data Availability Statement

The datasets presented in this article are not readily available because the SCImago group annually receives a raw data copy in XML format through a contract with Elsevier. The SCImago group has the possibility of downloading a copy of Dimensions in Json format through an agreement with Digital Science. We are not allowed to redistribute the Scopus and Dimensions data used in this paper. Requests to access the datasets should be directed to felix.moya@scimago.es.

## Author Contributions

VG-B: conception, data curation, and writing. AM: data curation. ZC-R: conception, data analysis, and writing. FM-A: conception, data analysis, and writing. All authors read and approved the final manuscript.

## Conflict of Interest

The authors declare that the research was conducted in the absence of any commercial or financial relationships that could be construed as a potential conflict of interest.

## Footnotes

^1^In our case, we subtract the Levenshtein distance (multiplied by 1.3) from the number of characters in the largest of the fields to be compared, thus obtaining a number indicative of the number of matching characters between the fields (with a 30% penalty). Recall that the Levenshtein distance is the minimum number of single-character edits (insertions, deletions, or substitutions) required to change one string into the other.

^2^ https://www.grid.ac/pages/policies
